# Methodological Considerations in N-of-1 Trials of Traditional Chinese Medicine

**DOI:** 10.1155/2021/6634134

**Published:** 2021-06-23

**Authors:** Haiyin Huang, Jiaqi An, Lizhi Lu, Mingli Wang, Huijia Chen, Xinlin Chen, Lihua Shen

**Affiliations:** ^1^Yueyang Hospital of Integrated Traditional Chinese and Western Medicine, Shanghai University of Traditional Chinese Medicine, Shanghai 200437, China; ^2^Basic Medical College of Guangzhou University of Traditional Chinese Medicine, Guangzhou 510006, China

## Abstract

More and more scholars choose N-of-1 trials for TCM clinical research. However, the quality of the experimental designs was uneven. Accumulating more than eight years of experience in exploring the N-of-1 trials of TCM, the authors and their team searched the related literature in main Chinese and English databases, referenced to relevant Chinese and international guidelines. The design, implementation, and data analysis of N-of-1 trials of TCM are still in in-depth exploration and practice. “Carryover effect” may affect the design and quality of the trials. Individualized treatment should be guided by the classic theories of TCM. It is expected to formulate reasonable observation periods and pairs and closely integrate individual and group statistical analysis.

## 1. Introduction

In the context of making decisions about an individual patient's care, N-of-1 trials have been considered to be among the most relevant and rigorous study designs for assessing treatment efficacy; they are listed as “level 1” evidence in the Oxford Centre for Evidence-Based Medicine 2011 levels of evidence [1]. As with crossover trials, N-of-1 trials eliminate confounding by covariates since each patient serves as his or her own control. The use of multiple crossovers within well designed N-of-1 trials increases confidence in the reliability of the results [2]. With the establishment and publication of CONSORT extension for reporting N-of-1 trials (CENT) in 2015, and the formulation of CENT for TCM in 2019 [3], N-of-1 trials are attracting more and more attention from scholars in China and around the world, and its application scope is also expanding.

We have cooperated with scholars from the Department of Clinical Epidemiology and Biostatistics, McMaster University (pioneer of N-of-1 trials), to explore the methodology of N-of-1 trials of TCM since 2012. This method is welcomed by patients because it embodies individual thinking and is close to the clinical practice of TCM. However, there are some problems in the current N-of-1 trials methodology of traditional Chinese medicine. We searched the published literature of N-of-1 trials of traditional Chinese medicine in major Chinese and international databases and want to summarize the rules and find out the corresponding countermeasures.

## 2. Methods

### 2.1. Eligibility Criteria

We included studies that fulfilled all of the following criteria: (1) journal articles published in English or Chinese, (2) the trial design included at least 2 trial cycles (pairs), and (3) studies or protocols of N-of-1 trails related to TCM.

### 2.2. Exclusion Criteria

Studies or protocols that met any of the following criteria were excluded: (1) meeting abstract, review, letter, commentary, editorial, book, or pamphlet, (2) studies that did not meet the design of N-of-1 trails, and (3) duplicate publication.

### 2.3. Search Strategy

We retrieved N-of-1 trials related to TCM published in English or Chinese in journals indexed by PubMed, Web of Science, the Cochrane Library, CNKI (China National Knowledge Infrastructure), China Biology Medicine (CBM), WANFANG MED DATA, and China Science and Technology Journal Database (VIP) and used “Single-patient trials,” “N-of-1,” “single case,” “individual patient and randomized,” “Single-Case Experimental Design (SCED),” “TCM,” or “CM” as the search terms. The searching was from the inception of the databases to October 2020. We came up with two main retrieval methods to improve the breadth and accuracy of retrieval.

Retrieval Method 1: Chinese database was retrieved by “N-of-1 trails” OR “single-case,” and then literatures related to TCM were manually screened out from the results. Due to the small number of N-of-1 trials literatures, most of which are published in the field of traditional Chinese medicine and have good relevance to traditional Chinese medicine, this method is indeed feasible.

Retrieval Method 2: retrieve English database by “Traditional Chinese Medicine AND N-of-1.” In the English database, N-of-1 trials literatures have been published in many fields and are not highly relevant to the field of traditional Chinese medicine. This method is adopted in the English database, and a single word (such as “N-of-1”) is used to search to improve the detection rate of literatures and avoid omission.

### 2.4. Study Selection and Data Abstraction

Teams of two reviewers, working in duplicate, independently screened titles and abstracts of all citations identified in our search. We obtained the full text of all articles that either reviewer deemed as potentially eligible. We assessed the articles according to CENT 2015 and CENT for TCM [2, 3], combined with 8-year experience of our team in the exploration of N-of-1 trials for TCM. We resolved disagreement through discussion and, when unsuccessful, with the help of a third author.

### 2.5. Data Analysis

We summarized the categorical variables with numbers and percentages for all analyses.

## 3. Results

### 3.1. Screening Process of the Research

**Figure 1:**
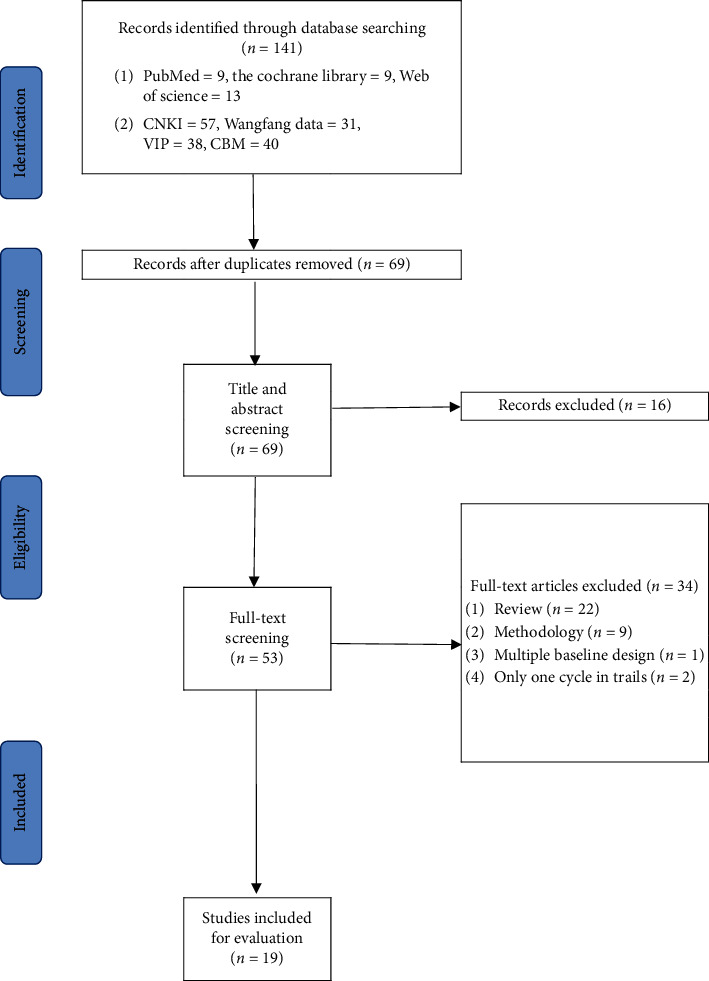
Search flowchart. CNKI, China National Knowledge Infrastructure; VIP, China Science and Technology Journal Database; CBM, China Biology Medicine.

Among 141 identified citations, 53 articles were retrieved for full-text screening. We included 22 research articles related to the N-of-1 trails of TCM. Of the 22 clinical reports, 2 of the trials were designed for only one cycle (pair) and could not be strictly classified as the N-of-1 trials, and 1 trial is indeed multiple baseline design (MBD), so all of the 3 trials were removed. Finally, a total of 19 articles proved eligible (Figure 1).

### 3.2. General Characteristics of Included Studies or Protocols

The first report of N-of-1 trials of TCM was published in 2010 [4], and 14 articles (73.68%) were published in recent 5 years. Among the 19 included papers, a total of 11 diseases were involved. Two papers did not report specific disease (the research object was TCM syndrome), and the TCM dosage forms were mainly decoction and capsule. The number of cases in each study was at least 1 and at most 50. Most of the results showed that N-of-1 trials of TCM are feasible and reflect the advantages of individualized treatment.

### 3.3. The Reason and Purpose of Carrying Out N-of-1 Trials in TCM Research

The reason and purpose of carrying out N-of-1 trials in TCM research are as follows: (1) most of the authors believe that N-of-1 trials can give full play to the characteristics of TCM individualized treatment (treatment based on syndrome differentiation) and embody the patient-centered trial design, so it is suitable for TCM clinical efficacy evaluation; (2) to evaluate the efficacy of TCM syndromes [5]; (3) to study the dose-effect relationship of Bezoar antihypertensive capsules and explore the individualized diagnosis, treatment, and evaluation of TCM [4]; (4) by comparing the efficacy of TCM syndrome differentiation with fixed prescription, to evaluate the feasibility of the N-of-1 trials of TCM and explore the individualized treatment evaluation method suitable for TCM research [6–9]; (5) to provide evidence for guiding doctors and patients to rationally administer Chinese patent medicine [10]; (6) some scholars think that N-of-1 trials need smaller sample size, easy to carry out with flexible design, so it can be a good complement to large randomized clinical studies [11]; (7) trying to solve the controversy of TCM clinic practice [6].

### 3.4. The Design of N-of-1 Trials of TCM

#### 3.4.1. Run-In Period

A run-in period occurs before a trial begins and is typically used to initiate trial medications (for example, preliminary observation on the prescription, dosage, and effect of TCM), determine tolerability, assess potential compliance with study regimens, identify adverse effects in a timely manner, or allow for washout of medication effects a participant was taking before formal enrolment in the trial [2, 12]. As traditional Chinese medicine is a kind of the mixture of herbs, its half-life period is difficult to determine biochemically. Professor Gordon Guyatt suggested that preliminary trials should be conducted to determine the washout periods along with the clinical experience of the researchers. Taking the changes of the patients' self-rated symptom scores as the main outcome, preliminary trials can obtain the onset time after administration and the efficacy maintenance time after drug withdrawal, so as to determine the observation period and washout period [8, 13]. This approach has been adopted by several authors [6–9, 14, 15].

Run-in period is particularly important for N-of-1 trials of TCM. However, only 4 of the 19 literatures contain preliminary trials, accounting for 21.05% of the included literatures. Therefore, the setting of run-in period has not received enough attention in N-of-1 trials of TCM.

#### 3.4.2. Washout Period

A washout period may occur between treatments to allow the effects of one treatment to wear off before proceeding with the next (that is, to reduce carryover effect) [16]. The length of washout period is generally determined by consulting the half-life of the medication. Since it is difficult to determine the process of TCM metabolism and half-life period, relatively reasonable washout period is defined mainly by the following two methods:

1. Conducting preliminary trial and combining the clinical experience of the researchers to determine a relatively reasonable washout period [8, 13] (see Section 3.4.1 of this article).
2. To determine the length of washout period by known half-life of the main active components of TCM or TCM compound. For example, Li et al. determined the one-week washout period by the known half-life of the active components of Semen Cuscutae and astragalus membranaceus [17].

There are some ethical problems in setting washout period when patients cannot receive active treatment. If the time is too long, it will be difficult to be accepted by the subject. There were two solutions: (1) not stopping the medication between the two periods, while the outcomes were measured in the end of each period, and the time (days or weeks) before the day(s) of outcome measure was supposed to be the washout period [7, 13]. (2) Chen et al. proposed the N-of-1 trials without washout period and their corresponding mixed effect models. The detection of carryover effect with the help of these mixed effect models is helpful to correct the influence of individual factors and period effects and compare the effects of the two kinds of interventions [16, 18].

Zi et al. [6] demonstrated the trial design of stopping medication for one week between the two treatments of TCM, which was originally aimed at extending the washout period of the former medication. Unexpectedly, it was welcomed and affirmed by most subjects, as their symptoms did not rebound during the week of stopping medication, and it was comfortable for the stomach.

#### 3.4.3. Length of the Observation Period and Pair (Cycle)

There were 10 trials involving three pairs [6, 8, 9, 14, 19–24] (the partial list is shown in Table 1). The maximum length of each period was 8 weeks [11, 20], most were 4 weeks (taking medicine for 3 weeks and stopping medication for 1 week) [4, 6, 8–10, 19, 21, 23]; 1 study was 3 weeks (taking medicine for 2 weeks and stopping medication for 1 week) [14]; 2 trials had 2 pairs (each pair contained two periods of 14 days each) [15, 25]. Some of the trials had long periods with the length of one pair up to 18 weeks [17]. Long trial time may increase the interference of unpredictable factors and decrease the subject's compliance. Most researchers selected four weeks as the length of the (observation) period.

#### 3.4.4. Randomization and Blinding

Randomization and blinding are important principles of N-of-1 trials. Randomization is mainly carried out on several pairs of cross-sectional trials of the same N-of-1 trial and has been implemented in almost all the N-of-1 trials of TCM. However, three N-of-1 trials failed to be blinded [22–24]. Even though some literatures reported the application of blinding, the reports of implementation method were not complete and transparent [21, 23, 25]. The forms of TCM include decoction, granule, and capsule. Due to the unique perception, taste, and smell of traditional Chinese medicine, it is extremely difficult to find a control drug that is exactly the same as the test drug [3, 26]. It is especially difficult for decoction or granule to make its control (simulation agent). In the N-of-1 trials where the same patient alternates between trial and control drugs, requirements for the biofidelity of the simulated (control) drug are higher than in a parallel randomized controlled trial. Haiyin Huang et al. reported in their study that the two traditional Chinese medicine decoctions may be similar in appearance and size, but there may still be subtle differences in taste and smell. To overcome the interference of this difference, they told the participants that the test and control decoctions may be effective regardless of taste and smell. Even if there are differences, most participants did not know which formula they were assigned to because they did not show a preference for a certain decoction [6, 7].

### 3.5. Choice of Treatment and Control

#### 3.5.1. Individualized Treatment

On the basis of disease differentiation, TCM researchers can carry out highly individualized treatment based on syndrome differentiation according to patients' different TCM syndromes. A total of 8 literatures [6–9, 13, 21, 22, 24] set the treatment based on syndrome differentiation as the treatment scheme in the trials. It not only met the requirements of N-of-1 trials but also retained the characteristics of TCM treatment based on syndrome differentiation. Zhang et al. [21] studied the therapeutic effect of TCM on postoperative patients with hypertensive cerebral hemorrhage according to the different syndromes of the four patients. For example, one case was diagnosed as hyperactivity of Liver Yang Syndrome and was given Tianma Gouteng decoction. In another case, purgatory phlegm decoction was used for phlegm dampness Mengshen syndrome, and later it was changed into Shenfu decoction. Placebo was used as the control for all of the four cases. Huang et al. [7] stipulated in the TCM medication program that the treatment based on syndrome differentiation can not only be performed individually but also be adjusted according to the patient's condition and syndrome changes during the whole process of N-of-1 trial while the control drug is always fixed. It conforms to the principle of “applying proper therapeutic measure in line with season, local conditions, and individuality” in TCM. In a series of N-of-1 trials, the different formulations may have different therapeutic effects, which presents new challenges for data integration analysis.

#### 3.5.2. Fixed Prescription for the Treatment of the Same TCM Syndromes

According to the purpose and requirements of the study, this format was adopted in three clinical reports: Huang [13] included subjects clinically diagnosed as kidney Yin deficiency syndrome and observed the efficacy of Wang et al.'s decoction in the treatment of kidney Yin deficiency syndrome, which can be regarded as a type of TCM syndrome differentiation and treatment. Hui Wang compared the efficacy of two different doses of Bezoar antihypertensive capsules in patients with the same syndrome type (hyperactivity of liver fire type) [4]. Liu compared the effect of Liuwei Dihuang capsule and placebo on TCM syndrome in patients with the same syndrome type (liver-kidney Yin deficiency syndrome) [15].

#### 3.5.3. Selection of the Control

The selection of appropriate control based on the research direction is crucial for N-of-1 trials. At present, there are mainly the following kinds of controls used in the N-of-1 trials of TCM:

*(1) Placebo Control*. Based on the analysis of the included literatures, it was found that few scholars chose placebo as the comparison of decoctions, which may be related to the simulation difficulty of TCM decoction. Sun et al. [20] selected the placebo with the taste, smell, and appearance most similar to ginkgo drop pills as the control. Liu et al. [19] used lactose, edible pigments, and bitters and added 10% experimental medicine as the placebo control for the granules of treatment based on syndrome differentiation. Liu et al. [15] selected the black kerneled rice and prepared *Rehmannia* root (10 : 1 ratio) as the control of Liuwei Dihuang capsule. At present, it is difficult for TCM placebo to simulate the taste and smell perfectly. Some scholars have reduced the difficulty of TCM placebo simulation through the choice of dosage form. For example, Yuhong [10] chose the soft capsule from among the Liuwei Dihuang honey bolus, water pill, liquid, soft capsule, and other dosage forms in order to make it easier for the placebo to imitate the experimental drug.

*(2) Related TCM Prescription as the Control*. It is mainly used for the control of TCM decoction. Xue et al. [8] selected *Bronchiectasis Stabilization Decoction* (fixed formula) as the comparison of treatment based on syndrome differentiation. Zi et al. [6] selected the individualized decoction removed of heat-clearing TCM as the control of the individualized decoction in the N-of-1 trials on the treatment of bronchiectasis and studied the role of heat-clearing TCM in the stable period of bronchiectasis. Li et al. [17] used the prescription removed of the main effective drugs (astragalus membranous, semen Cuscutae) as the control.

*(3) Conventional Basic (Western Medicine) Treatment*. As a control, conventional basic treatment is mostly used to compare the efficacies of TCM treatment and Western medicine treatment in the same situation, or the efficacies of a single treatment regimen and a combination of treatment regimen. Yu et al. [24] chose the conventional basic treatment as the control of the prescription based on syndrome differentiation without blinding. Jiao et al. [22] used telmisartan as the control in the N-of-1 trials to observe the treatment of IgA diabetic nephropathy with Modified Huangqi Chifeng Decoction combined with telmisartan.

Some combined application schemes of TCM and Western medicine adopted double-blind and double-simulation form, indicating that the degree of blinding in some N-of-1 trials for TCM has reached a relatively high level [14, 21].

### 3.6. Outcome Measures

#### 3.6.1. Selection of Outcome Measures

The characteristics of the N-of-1 trials are to fully respect the choice of the patient, to determine the main problems to be solved jointly by the doctor and the patient. Almost all of the included reports took the clinical symptom score that the patient is most concerned with as an important outcome. Some studies used objective signs, such as blood pressure at a given time [4] and 24-hour sputum volume [6–9, 13]. Some studies used laboratory indicators, such as serum IL-6 [21], 24h urinary protein quantification [22], blood routine [14], serum creatinine (SCr), and creatinine clearance rate (Ccr) [24].

Safety outcomes include regular observation of adverse events related to the trials, measurement of blood and urine routine, liver and kidney function, and electrocardiogram, if necessary, terminating the experiment and unblinding. Good outcomes should be easy to repeat measurement, sensitive, and generally recognized as effective.

#### 3.6.2. Minimal Clinically Important Difference (MCID)

The most direct outcomes that best reflect the needs of patients are the clinical symptoms which trouble patients (such as cough, wheezing, loss of appetite, and insomnia) and the quality of life scale. The 7-point Likert scale recommended by Professor Guyatt, in which the patients used a diary form to self-evaluate the severity of the symptoms which they were concerned about (usually 4 to 8 symptoms) on a daily basis, was more intuitive when supplemented by visual analogue scales (VAS). Its significant advantage is that its reliability and its MCID have been fully proofed before its application. In the 7-point Likert scale, an average difference of 0.5 was defined as the minimum clinically important difference (MCID) [27, 28]. Three included literatures have been found to have adopted the 7-point Likert scale as an important outcome [6–8]. Two articles used either a 6-point Likert scale or a 5-point Likert scale [10, 15]. Some authors used some widely accepted scales [20, 25]. Some authors adopted TCM syndrome score [9, 19, 22]. The results of these scales or scores may be statistically significant but not clinically significant; therefore, in addition to reporting statistical differences, it is also necessary to explain whether the score or scale has demonstrated MCID and whether the difference has reached MCID. Besides, MCID is essential for converting quantitative outcomes into binary outcomes such as “effective/response rate.”

#### 3.6.3. Negative and Positive Results

Because N-of-1 trials have the scientific design of randomization and blinding, which reduces the selection bias, and the small sample size increases the probability of false negatives, the probability of negative results will inevitably increase. Among the results of the 19 TCM N-of-1 trials retrieved in this study, 3 were negative, or even though, there is a statistical difference (*P* < 0.05), but not clinically significant [4, 6, 10]. It should be recognized that a negative result from a high-quality N-of-1 trial is no less important than a positive result. For example, Yuhong et al. evaluated the clinical efficacy of Liuwei Dihuang soft capsule in the treatment of 50 patients with kidney Yin deficiency syndrome by a series of N-of-1 trials. The meta-analysis results showed that Liuwei Dihuang soft capsule and placebo had no difference in the efficacy of improving kidney Yin deficiency syndrome (*P* > 0.05). After the trials, the drug was discontinued in the nonresponders, and its positive significance was to avoid the abuse of patent Chinese medicines [10]. When interpreting negative results, we must first realize that negative results are as valuable as positive results and then make judgments based on the doctor's clinical experience.

### 3.7. Selection of Statistical Methods

In the early years, N-of-1 trials were mainly the self-control of individual cases, and the statistics of repeated crossover data in multiple rounds (pairs). With the development of the N-of-1 trials methodology, it was found that a series of N-of-1 trials can summarize the overall treatment effect of a group and obtain relevant treatment information for individual participants [29, 30]. The ideal statistics of a series of N-of-1 trials should be the combination of individual statistics and group statistics: to extend the application of this method from a single case to the combination of individuals and groups. At the same time, it also makes it possible for clinical trials of TCM to treat chronic diseases to generally adopt a series of N-of-1 trials in the future.

CENT 2015 encourages the use of confidence intervals which is often possible for the analysis of important clinical differences [2]. However, the Chinese literatures for N-of-1 trials did not pay enough attention to this, and only a part of the literatures published confidence interval.

#### 3.7.1. Statistics of Individual Data

The most attractive aspect of N-of-1 trials is that, by the comparison of two treatments for the individual patient, a hypothetical or potentially more effective prescription or treatment can be screened out. By repeating N-of-1 trials on the same patient, we can continually improve the effectiveness of the prescription or treatment for each individual. This is the ultimate goal of the research on individualized treatment (syndrome differentiation) by TCM [7]. However, statistical analysis of individual cases was not performed in 9 included articles [9, 14, 17, 19–21, 23–25], accounting for 47.36% of the included literature, which is a pity for the evaluation of individualized treatment of TCM. If the data were normally distributed, paired *t* test was preferred for single case statistical analysis. Paired Wilcoxon signed rank tests were conducted to analyze the data which were not normally distributed. Although paired *t* test was used in many literatures [6, 7, 11, 22], some N-of-1 studies also used *t* test (its statistical power is lower than paired *t* test) [25]. Since the same subject receives multiple treatments and measurements at different times, it is called repeated measurement data and has autocorrelation interference to the results. Guyatt's strategy was to average the data in a certain period of time (for example, averaging the data of 7 days as the average value of the week) and then perform statistical analysis [7, 27]. The data published in some literatures did not clearly indicate the time and frequency of measurements, which needs to be improved [21, 22].

#### 3.7.2. Population Statistics for a Series of N-of-1 Trials

Commonly used statistical methods include meta-analysis, hierarchical Bayesian statistical method, or mixed effects model analysis [31]. However, hierarchical Bayesian statistical method has not been used in the N-of-1 trials for TCM. Among them, meta-analysis [4, 10, 15] was used in three articles, *t* test or paired Wilcoxon signed rank tests in 5 articles [8, 13, 22, 24, 26], and mixed effects model in 3 articles [6, 7, 11].

#### 3.7.3. The “Carryover Effect” of TCM

**Figure 2:**
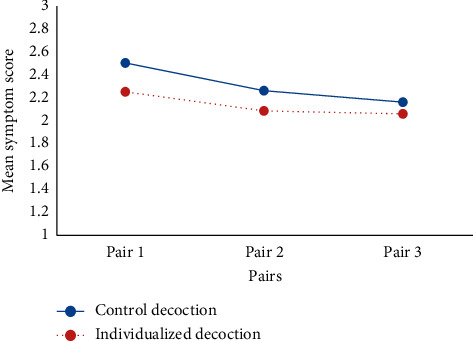
The gap between the mean symptom scores of the individualized decoction and control decoction had a diminishing trend from first to third pairs in a series (14 cases) of N-of-1 trials, suggesting the possibility of “carryover effects.” Excerpted from “Huang Haiyin, Yang Peilan, and Wang Jie et al.'s Investigation into the Individualized Treatment of Traditional Chinese Medicine through a Series of N-of-1 Trials. Evid Based Complement Alternat Med. 2018; 2018 : 5813767. doi: 10.1155/2018/5813767.”

CENT2015 requires the description of the statistical methods used to account for carryover effect, period effects, and intrasubject correlation. Only 3 articles have given some explanation on this requirement [6, 11, 15]. There are two articles mentioning that the “carryover effects” of traditional Chinese medicine may affect the results. Yuhong [10] speculated that a limitation of their study was washout period which has not been fully considered, which resulted in “carryover effects” of Liuwei Dihuang decoction (LDD) interfering with the differences between LDD and placebo. Huang et al. [7] discussed that “the nature of traditional Chinese medicine might not meet with a certain requirement of the classic N-of-1 trials perfectly: the treatment should have a rapid onset and stop acting soon after it is discontinued.” They found that “the gap between the mean symptom scores of the individualized decoction and control decoction had a diminishing trend from first to third pairs in a series (14 cases) of N-of-1 trials (Figure 2), suggesting the possibility of “carryover effects.””

### 3.8. Follow-Up after the Trial

Only a few literatures have mentioned whether the treatment plan of the subject has changed, or whether there were long-term adverse reactions after the completion of the trials [10]. It is worthy of attention in future research.

### 3.9. Main Findings

Since the publication of the first N-of-1 trials of TCM [4], the practitioners of TCM and integrated TCM and Western medicine have made valuable explorations on the feasibility and rules of N-of-1 trials of TCM over the past 10 years. Hui Wang et al. proved that the high dose of Bezoar antihypertensive capsules could reduce the systolic blood pressure with the syndrome type of hyperactivity of liver fire. N-of-1 trials can be used as a management tool for clinical medical practice and preliminary exploratory research on new drugs [4]. Huang et al. [13] proposed a method to establish a relatively reasonable washout period through the run-in period of N-of-1 trials for TCM. Yuhong et al. [10] used the results of a series of N-of-1 trials to make the nonresponders stop the drug after the trials. The positive significance is to avoid the abuse of Liuwei Dihuang capsule. Liu et al. [5] found that the N-of-1 trials could be used to evaluate the efficacy of the treatment based on syndrome differentiation to improve TCM syndromes, and the results were reliable. Chen et al. [16] proposed a design of N-of-1 trials without washout period and its corresponding mixed effect model, which could be used to detect the carryover effect of TCM. Finally, the publication of CENT for TCM in 2019 [3] showed that the national TCM community have attached great importance to this individualized clinical trial method.

## 4. Discussion

In summary, N-of-1 trials of TCM have made great progress in the past decade. However, we still face many difficulties, such as certain difficulties in the implementation of blind methods, sometimes the selection of outcome measures was unsatisfactory, and the statistical methods were not standardized, etc. There are other problems that manifested in the following aspects.

### 4.1. Existing Problems

#### 4.1.1. The Contradiction between Treating Symptoms and Curing Root Causes

TCM treatment emphasizes that “the cure must be based on the etiology,” rather than simply eliminating symptoms. This was the case with one of the core principles of naturopathic medicine: “Treat the Cause (tolle causam)” [32]. Although N-of-1 trials embody a high degree of individualization and are close to the clinical practice of TCM, they are most suitable for treating symptoms, especially chronic symptoms, rather than treating the root cause.

#### 4.1.2. The Interference of “Carryover Effects”

N-of-1 trials generally require that the studied drugs have the characteristics of fast onset, short half-life, and fast disappearance after stopping use [2]. The components of TCM are complex and the onset and expiration time (half-life) are relatively long, so it might not meet these requirements. “Carryover effects” may be an unfavorable factor affecting the design and quality of N-of-1 trials of TCM [7, 10, 16].

#### 4.1.3. The Influence of Seasons and Time Rhythms

In addition to individualized treatment, traditional Chinese medicine believes that people's physiological and pathological changes will inevitably be affected by seasons, climate, and time rhythms. Therefore, the occurrence of diseases and the principles of health preservation and treatment are different in different seasons. In the treatment of chronic diseases, the patient's TCM syndrome type may also change. Therefore, in the N-of-1 trials of TCM, individualized prescriptions should also change according to the situation. Although Huang et al. [7] stipulated in the TCM medication program that the treatment based on syndrome differentiation can not only be performed individually but also be adjusted according to the patient's condition and syndrome changes during the entire process of the N-of-1 trial, how to overcome the influence of seasons and time rhythms in the N-of-1 trials of TCM is still challenging.

### 4.2. Development Trends

In recent years, hierarchical Bayesian statistical method has become one of the main statistical methods for a series of N-of-1 trials with remarkable characteristics [2, 29, 30, 33]. Some scholars discussed in detail the advantages of Bayesian method over general statistical methods, mainly including the following: (1) both individual and aggregate analyses can be simultaneously and coherently undertaken. (2) It is easy to introduce confounding variables [34–36], such as the gene type or different TCM syndrome types of different patients, the severity of bronchiectasis, sputum culture (whether *Pseudomonas aeruginosa* is positive), and the potential carryover effect of TCM. Regarding the “carryover effect,” it is envisaged to adopt the mixed effect model proposed by Chen et al., which was supposed to detect the “carryover effect” [16, 18]. (3) A prior information can be introduced. Prior information, sample information, and posterior information are the three important types of information of the Bayesian model. The prior information comes from previous research. The sample information comes from the existing data, and the Bayesian model uses the sample information to update the prior information to obtain more reliable posterior information. In the hierarchical Bayesian model, previous data and new experimental data belong to a continuous data chain. When new experimental data is generated, the data chain is updated to produce more accurate posterior information [33]. Though having not been used in N-of-1 trials of TCM, hierarchical Bayesian statistical method is worthy of studying and promoting and is expected to improve the reliability and sensitivity of N-of-1 trials of TCM.

## 5. Conclusion

In short, the design, implementation, and data analysis of N-of-1 trials of TCM are still in in-depth exploration and practice. “Carryover effect” may affect the design and quality of N-of-1 trials of TCM. Individualized treatment should be guided by the classic theories of TCM. It is expected to formulate reasonable observation periods and pairs and closely integrate individual and group statistical analysis.

## Figures and Tables

**Table 1:** Characteristics of some representative N-of-1 trials of TCM.

Author	Reasons for conducting N-of-1 trials	*n*	Run-in period	Treatment	Control	Pairs	Observation period	Randomization and blinding	Primary outcome measures	Statistical methods	Conclusion
Yuhong et al. 2013 [10]	Providing evidence for rational use of LDD.	50	No	LDD	Placebo	3	4 weeks	Yes	Likert scale, individual completion rate, response rate, and posttrial decision-making	Individuals: the self-designed criteria; population: meta-analysis	Nonresponders ceased the LDD. The positive significance is to avoid the unreasonable drugs use.
Huang et al. 2018 [7]	Exploring the establishment of clinical efficacy evaluation methods in line with the characteristics of individualized diagnosis and treatment of TCM.	17	Yes	Individualized herbal decoction	Standard decoction for stable bronchiectasis	3	4 weeks	Yes	7-point Likert scale	Individuals: paired *t* test; population: mixed effects mode	Optimizing the combined analysis of individual and group data, the improvement of statistical models may make contribution in establishing a method of evaluating clinical efficacy in line with the characteristics of TCM.
Wang et al. 2010 [4]	To study the dose-effect relationship of Bezoar antihypertensive capsules and explore the individualized diagnosis, treatment, and evaluation of TCM.	11	No	High-dose Bezoar antihypertensive capsules	Low-dose Bezoar antihypertensive capsules	3	4 weeks	Yes	Blood pressure, TCM symptom score	Paired *t* test, meta-analysis	Bezoar antihypertensive capsule can be used for mild to moderate hypertension particularly for lowering systolic blood pressure.
Chen et al. 2020 [11]	N-of-1 trials can provide more flexible clinical trial design for TCM and require a smaller sample size.	10	No	Modified SJZD with mesalazine placebo	Mesalazine with SJZD placebo	3	8 weeks	Yes	Visual analogue scale (VAS) of symptom score	Mixed effects mode	This article is a protocol and there is no conclusion.
Liu et al. 2018 [15]	To evaluate the efficacy of TCM syndromes by N-of-1 trials.	24	Yes	Liuwei Dihuang capsule	Placebo	2	2 weeks	Yes	5-point Likert scale	*t* test, ANOVA for repeated measurement, and meta-analysis	N-of-1 trials can be used to evaluate the therapeutic effects of TCM syndromes.
Zhang et al. 2012 [21]	N-of-1 trials enable scientific evaluation of the individualized diagnosis and treatment of TCM.	4	No	Individualized herbal decoction and basic treatment	Basic treatment and Chinese medicinal decoction placebo	3	4 weeks	Yes	TCM symptom score, serum IL-6	*t* test	It is feasible to apply N-of-1 trials in clinical research of TCM.
Wang et al. 2016 [9]	To evaluate the feasibility of N-of-1 trails of TCM.	1	Yes	Individualized herbal decoction	Standard decoction for stable bronchiectasis	3	4 weeks	Yes	7-point Likert scale	Paired *t* test	N-of-1 trails reflected the advantage of TCM individualized treatment in this patient, providing with the highest rank of evidence for the patient.
Yu et al. 2012 [24]	Individualized treatment of TCM is the unique advantage.	3	No	Individualized herbal decoction	The basic treatment	3	4 weeks	Randomization but no blinding	TCM symptom score, SCr, and Ccr	*t* test	N-of-1 trails for the clinical studies of TCM are useful and feasible.

Abbreviations: TCM, traditional Chinese medicine; LDD, Liuwei Dihuang decoction; ANOVA, analysis of variance; SJZD, Sijunzi Decoction; SCr, serum creatinine; Ccr, creatinine clearance rate.
